# Current Stereotypes Associated with Nursing and Nursing Professionals: An Integrative Review

**DOI:** 10.3390/ijerph19137640

**Published:** 2022-06-22

**Authors:** Cristina Teresa-Morales, Margarita Rodríguez-Pérez, Miriam Araujo-Hernández, Carmen Feria-Ramírez

**Affiliations:** Nursing Department Teaching and Research, University of Huelva, 21004 Huelva, Spain; cristina.teresa@denf.uhu.es (C.T.-M.); miriam.araujo@denf.uhu.es (M.A.-H.); carmen.feria@denf.uhu.es (C.F.-R.)

**Keywords:** literature review, male nurses, nurses, nursing, stereotyping

## Abstract

Nursing and nursing professionals are associated with social stereotypes, which may hinder the profession’s development and future prospects as a scientific discipline. The aim of this study was to identify and describe the stereotypes associated with the nursing profession—students and professionals. Therefore, we carried out an integrative review. The search was conducted using PubMed, WOS, and CINAHL databases, and its search strategy was based on a combination of standardised keywords and natural vocabulary, with a temporal limit between 2016 and 2021. The data extraction and analysis was based on the conceptual framework developed by Whittemore and Knafl. Twenty-seven studies were included in the review, and their results were classified and coded. Two categories emerged, namely, stereotypes relating to the professionals’ gender and stereotypes relating to the profession itself. We concluded that the nursing profession is viewed as female with low skills, social status, salary, academic level and entry requirements, and with little autonomy. Male nurses’ professional competencies and masculinity are questioned, while the work carried out by female nurses is viewed as unprofessional. To reduce these stereotypes and bias we must present the nursing profession as a scientific discipline, developed by both men and women. Specific channels for this awareness-raising work include interventions from universities and the media, and participation in health policies.

## 1. Introduction

Stereotypes may be defined as a mental image of the characteristics and behaviours shared by a group of people. They are used to simplify more complex ideas accepted collectively by a group or society, and they are unchanging [1,2,3]. Stereotypes are closely linked to gender, which is a cultural construction that attributes different types of behaviour to men and women as a result of their sexual differentiation [4]. They are sociocultural constructions, defined and categorised according to what is considered “natural” or “proper” [3]. Male and female roles are stereotyped in different ways. These stereotypes are accepted by most societies and are perpetuated by education, the media, and within families [3,5,6], for example, creating a gender identity and a way of differentiating between the sexes. 

Throughout history, in almost every culture, women have been assigned a reproductive gender role based around biological reproduction and the home, involving raising children, feeding and caring for members of the household, and organising and maintaining the home [7,8]. Meanwhile, men are assigned a productive role revolving around income-generating activities and paid work to support their families; these are considered the main type of productive activity and are highly valued by society as a result [7,8]. These assigned gender roles constrain women and steer them towards traditional caring roles, keeping them away from the public sphere and from activities considered more appropriate to men, which are more highly paid and offer higher social status [9]. The categorisation of these behaviours has led to discrimination based on the value assigned to each role by society [10].

Professions and professionals develop within a social context shaped by stereotypes that influence perceptions of the profession and affect its growth and development. Nursing has always been associated with components of gender, which relate to the origins of the profession and gender roles more broadly [11]. Social perceptions of nursing professionals are thus shaped by the social divide between men and women, and by the roles attributed to women and nursing interventions in particular [12]. This vision influences nurses’ own perceptions and self-concept, even affecting their decision as to whether or not to pursue a nursing degree [13].

Previous studies have focused on the social image of the nursing profession [14,15,16], its representation in the media [17,18], the situation of male nurses within the profession [11,19,20] and the social perception of nurses [13]. According to these studies, at the end of the 20th century and beginning of the 21st century, the public image of nurses was diverse and incongruous [14]. The nursing profession was perceived as a profession little valued and understood by society [15,16], maybe because people had incorrect information about the functions performed by nursing professionals [17]. Therefore, this public image was predominantly based on misconceptions and stereotypes [13,14]. The media perpetuated the stereotypes of the nurse as angels of mercy, the doctor’s handmaiden and sexy nurse or sex object [14]. The male nurse was viewed as effeminate or homosexual [19] or he was erased from the image of the profession [18], making him invisible to society [20]. However, the social image of the nursing profession and its professionals changes with, or should change with, changes in society. At the social level, the last decade has been characterized by increased access to various sources of information, communications and social networks. On a professional level, the visibility of the nursing profession has grown with events such as the WHO’s declaration of the Year of the Nurse and the Midwife, in 2020 (Year of the Nurse and the Midwife 2020 (who.int (accessed on 20 June 2022))); the Nursing Now campaign from the International Council of Nursing (https://www.nursingnow.org/ (accessed on 20 June 2022)); the #FakeNurses campaign in Spain ((183) Campaña #FakeNurses #EsaNoSoyYo-YouTube), and the significant public exposure of healthcare professionals as a result of the COVID-19 crisis. These circumstances should have favoured access to a deeper knowledge about the nursing profession. We may thus expect that the gender stereotypes of nursing professionals and the lack of knowledge about the profession have been minimized. For these reasons, our research question was: “What are the stereotypes with which the nursing profession and nursing professionals are currently associated?” So, the identification of studies, which could provide evidence of stereotypes associated with the nursing profession and professionals, will allow that these stereotypes and elements that impacting on access to training and on professional development can be addressed. This review paves the way for interventions to tackle the origins, perpetuation, and consequences of stereotypes relating to the nursing profession to be planned.

## 2. Materials and Methods

Aim: to identify and describe the stereotypes associated with the nursing profession, both students and professionals.

Design: The stages followed in this integrative review are based on the methodological framework developed by Whittemore and Knafl [21]: identification of the research problem; literature search; data assessment; data analysis and synthesis; and presentation of selected data.

First stage: Identification of the Research Problem. This stage has been described in the Introduction section.

Second stage: Literature Search. The literature search was conducted between June 2021 and September 2021. The databases explored were: PubMed, WOS, and CINAHL. The search was limited to studies published between 2016 and 2021 in English, Spanish, and Portuguese. The inclusion criteria were: articles whose object of study was stereotypes relating to the nursing profession or nursing professionals (IC1) and original articles using a qualitative, quantitative, and/or mixed methodology (IC2). The exclusion criteria were: reviews and articles using a historical methodology (EC1); studies aiming to identify the stereotypes of other groups held by nurses (EC2); and methodological quality evaluation (EC3), which we will describe in Data Assessment. The search strategy was based on a combination of the following keywords: stereotyping/nursing/nurses/nurse. The thesaurus of each database was selected and used for the search using standardised terms (MESH for PubMed; MH for CINAHL). To complete the search, the same terms were used as keywords in natural language to search by title and abstract (T/A for PubMed and CINAHL; TI and AB for WOS). The details of the search strategies employed can be seen in Table 1.

**Figure 1:**
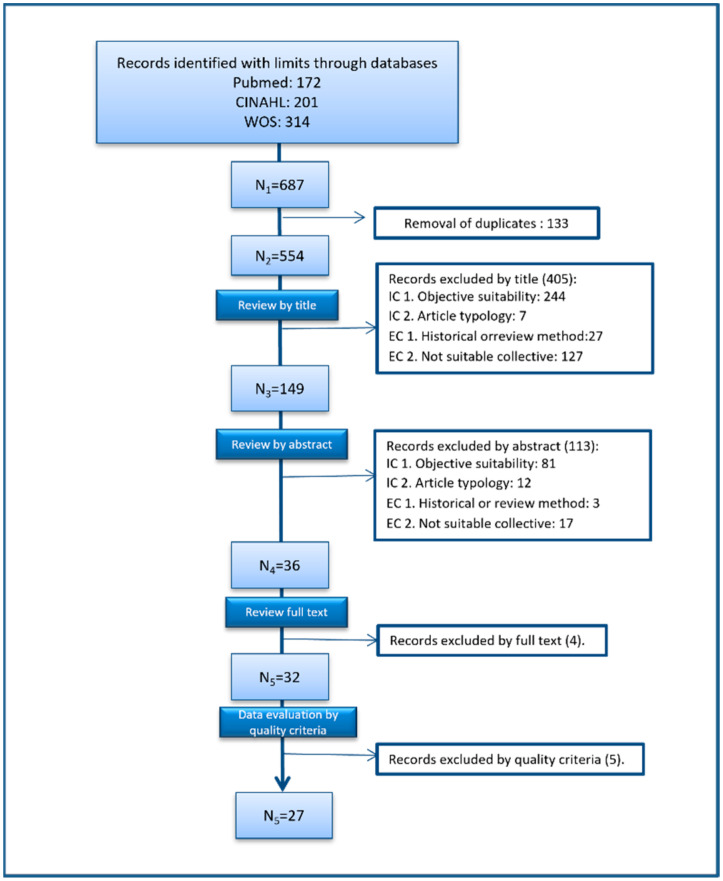
Flow diagram of the literature search and data assessment process.

We found 687 articles when the search limits were applied. Once duplicates were removed, there were a total of 554 articles remaining, which were analysed and screened for suitability by reading the titles (*n* = 146). During the abstracts review, inclusion and exclusion criteria were applied (*n* = 36). Another 4 articles were excluded during the full text review because their results were not associated with stereotypes of nursing or nurses (*n* = 32). (See Figure 1. Literature search and data assessment flow diagram.)

Third stage: Data Assessment. Following the Whittemore and Kanfl [21] recommendation, the methodological quality of studies was assessed. This was done using the MMAT [22], a tool consisting of two sections: a two-question section common to all studies and a specific five-question section for each type of study. The templates for quantitative, qualitative and mixed methods studies were reviewed independently by the researchers. Studies that did not pass the initial screening questions common to all types of study were rejected (five articles). Finally, 27 articles were reviewed. (See Figure 1. Literature search and aata assessment flow diagram.)

Fourth stage: Data Analysis and Synthesis. For the extraction and synthesis of data, researchers performed a comprehensive and exhaustive reading of the selected studies. The results of the selected studies were classified and coded via an inductive approach and the emerging categories were reduced, using the analytical framework developed by Whittemore and Knafl [21].

Ethical approval: The methodology used in this study does not require ethical approval.

## 3. Results

Descriptive analysis: 27 articles were included: 7 quantitative studies; 18 qualitative studies; and 2 mixed methods studies. Study samples included: young people and university students (no. 4); nursing students of both sexes (no. 5); male nursing students (no. 5); nurses of both sexes and professionals working with nurses in teams (no. 12); patients (no. 3); and the general public (no. 2). It should be noted that some studies had a sample composed of more than one population sector. Table 2 provides a summary of the studies included.

Qualitative analysis: the qualitative analysis of the results of the analysed articles produced two general themes: stereotypes relating to the professionals’ gender; and, stereotypes of the nursing profession. We show in Table 3 each theme with its associated sub-themes and categories.

### 3.1. Theme 1. Stereotypes Relating to Professionals’ Gender

In Kim and colleagues’ study [24], 48.8% of the participants held progressive views on nursing, pointing to an absence of gender stereotypes relating to the exercise of the profession and to the possession of appropriate skills enabling nursing professionals to perform adequately. They perceived professionals of both genders as equals and as necessary members of the healthcare team, who work equally hard at their jobs [24]. However, this perception is contradicted by the results obtained for the remainder of the study sample and by other authors, indicating that there are a number of stereotypes relating to professionals’ gender. These stereotypes are listed below:

#### 3.1.1. Subtheme 1.1. Female Nurses’ Stereotypes

C.1 Female Nurses: The Ideal Gender for Nursing

The respondents in Cottingham and colleagues’ paper [42] defined nursing as a profession that has traditionally been carried out mainly by women. This coincides with the 66.8% male and 72.4% female nursing and midwifery professionals interviewed in Stanley and colleagues’ study [49], echoing the results of numerous other studies [27,28,38].

The woman-nurse nexus is more pronounced in other studies, where nursing is viewed as a job aimed at women [41]; characteristic of women [40,43,46]; suitable only for women [39], and more appropriate for women [32]. This stereotype is identified by professionals themselves: 56% of men and 50.75% of women in Stanley and colleagues’ article [49]; male nurses [27]; and female nurses who feel that nursing is an appropriate, suitable profession for them [40].

The stereotype of women as innately suited to caring [26] can be seen in patients’ preferences for female nurses. This is corroborated by male student nurses [33]; 84.2% of the Spanish general public [46]; 85% of the Saudi university community [44]; and patients [27]. It is most commonly seen among female patients [26], who may oppose care from male nurses [39]. This preference may be related to the normalisation of women delivering personal care without sexual connotations [33]. By contrast, Rabie and colleagues [26] showed that some male patients prefer to receive care from male nurses as they feel more comfortable with them.

C.2 Female Nurses: Family Struggles

In Elmorshedy and colleagues’ study [44], 50% of the participants would be willing to marry a nurse, but 65% believed that being a nurse had a negative impact on women’s social lives and that nurses delayed marriage due to the demands of their profession. A similar trend emerged in another paper [37], where participants expressed the belief that women should interrupt or abandon their professional careers at certain points in their lives to look after their families. These beliefs may explain the lack of family support perceived by some women when they decide to become nurses [32].

#### 3.1.2. Subtheme 1.2. Male Nurses’ Stereotypes

C.3 Male Nurses: Out of Place

Social perceptions of nursing as a women’s job give rise to the notion that men are out of place in nursing or that they are unsuitable for the profession. This perception is expressed by: 16% of the general public [24]; patients of both sexes, who explain that male nurses do not reflect their expectations or understanding of the nursing profession [27]; clinical nurses [25] and nurse educators, who stated their belief that men should not be nurses [42]; and male student nurses’ families, who considered nursing to be a women’s profession [37]. This stereotype was echoed by male student nurses in the study by Juliff and colleagues [41], which found that society has a biased view of what nurses do and of how men fit into nursing roles.

Generally speaking, it is considered inappropriate for male nurses to work in gynaecology, obstetrics, maternity, and paediatric units [24,25,29,37,41,48], where professionals and/or patients did not permit or condone male students or professionals carrying out tasks such as gynaecological examinations and care for newborns and infants, as they were considered to be less skilled and suitable for these activities despite their training and prior experience [25,29]. Similar feelings were expressed with regard to procedures relating to women’s hygiene in these and other units [41,48].

Some studies concluded that men are associated with the following characteristics as carers: hesitant [42]; untrustworthy [27]; less sensitive to others’ emotions, or less able to express themselves than women [41,42]; disorganised, irresponsible, careless when delivering basic care, reckless, and disrespectful of confidentiality [26].

In contrast with these stereotyped beliefs about male nurses based on broader understandings of male roles, another vision is also expressed: male nurses are caring, open-minded professionals [42]; friendly, empathetic, helpful, punctual, enthusiastic, kind, and better at management than female nurses [26,29,33].

Men’s allegedly greater management and leadership skills are supported by multiple sources [28,29,45,49]. Some sources note that care and personnel management roles are earmarked for the minority of male nurses [28,29], who are considered and consider themselves as the elite of the profession [36].

When it comes to delivering care, men are considered and consider themselves to be better at performing techniques such as venous catheterisation [29] and administering intravenous medication and drugs [37,45]. The female student nurses surveyed by Carlsson and colleagues [45] believed that they were good at delivering basic care, communicating with patients, handling sensitive information, and providing comprehensive care coordination. Meanwhile, male student nurses perceived themselves as being good at administering drugs and applying pharmaceutical knowledge, handling medical devices, and responding to emergencies. Men appear to have a preference for more technical areas, such as emergency, critical care, and surgical units, as well as mental health and psychiatric units [29,37,41,49]. In another study [29], this preference is linked to the fact that these units are the least visible to the Arab public, which views male nurses as inadequate.

Finally, Stanley and colleagues [49] found that 50.6% of men and 40.4% of women did not agree that male nurses are unsuited to the profession. The item “Men make good nurses” came seventh and sixth among students in the first and third years of the nursing degree, respectively, in Čukljek and colleagues’ paper [47]. Meanwhile, male nurses’ ability to dedicate more time to professional development than female nurses due to their lesser family responsibilities was highlighted as a positive characteristic [27].

C.4 Male Nurses: Male Weakness or Homosexuality

In numerous studies, homosexuality was identified as one of the most common stereotypes relating to male nurses. Several studies confirm this belief among patients of both sexes [27,37]; among nursing professionals themselves [48], and student nurses’ families [41]. Patients were even observed to exhibit more introverted behaviour until they had confirmed that the male nurse caring for them was not homosexual [42]. However, another study dismissed this belief, and this stereotype was rejected by 73% of its participants [46].

A relationship between homosexuality and the decision to study nursing was identified [25], an idea that was held by students themselves [48]. This has an influence on men when deciding whether or not to enter the profession [38]. The sample in Stanley and colleagues’ study [49] mostly agreed with this statement (59.6% of men; 50.9% of women), leading to changes in male nurses’ masculinity in some cases [41].

Another factor that calls male nurses’ masculinity into question and provokes feelings of shame is the use of female terms such as ‘nurse’, ‘sister’, and ‘matron’ to refer to them. These social customs perpetuate perceptions of nursing as a women’s profession [29,34,36].

These days, social acceptance of different sexual orientations and a trend towards unisex roles allows men to display sensitivity, warmth, and affection without being labelled as homosexual, prompting this stereotype to be perceived differently [33,42]. There are also more extreme perceptions, whereby male nursing professionals are assumed to be aiming for senior management roles if they accept this feminisation [36]. In addition, some male nurses reject the existing model of masculinity [33].

C.5 Male Nurses: A Sexual Threat

Professional nursing care requires physical contact between nurses and patients. It is considered normal for female nurses to engage in non-sexual, personal physical contact with men viewed as unsuitable for this task [33,42]. This means that male nurses are avoided [24] or perceived as a heterosexual threat to women or a homosexual threat to men [27], which may even make them vulnerable to accusations of inappropriate behaviour towards patients or female colleagues [26,37,41]. It appears that male nurses’ gender is not always decoupled from their professional abilities, giving rise to misinterpretations of their actions or distrust in their intentions [27,32]. The clearest manifestation of this stereotype was seen in a female teacher addressing a male participant in Cottingham and colleagues’ paper [42], telling him that she would not assign a female patient to him because “she knew what he would do”.

C.6 Male Nurses: Shame and Embarrassment

Some nursing students were ashamed by their perceived rejection by society, their families, their friends, and other healthcare professionals when they expressed their desire to become nurses [48]. This rejection and the possibility or reality of being mocked [37,41] provoke shame in many male nurses, who opt to avoid telling people that they are nurses [23,31,35]; conceal their work using ambiguous terms such as ‘healthcare professional’ [31,41]; or hide in units where little contact with the public is required [29].

Another source of shame for male nurses is linked to issues with the provision of uniforms by healthcare facilities, which do not always have uniforms for men [25,26], obliging them to wear women’s uniforms. In some contexts, being a nurse can have a negative impact on men’s marriage prospects, as many people would refuse to allow their daughters to marry a nurse [23,29,35].

C.7 Male Nurses: Physical Strength

Numerous references to male nurses as the physical force behind the nursing profession were identified. On some occasions, this was perceived as an advantage when it was necessary to move or hold patients [23,24,26,49], while on other occasions, male nurses believed that they received greater recognition or were valued more for their physical strength than their professionalism [26,29,48]. This had a negative impact on their careers as their female colleagues received more opportunities for practical learning, while they were only required to perform tasks requiring physical strength [34].

C.8 Male Nurses: Should Be Doctors

The studies in this review highlighted the belief that men who choose to study or work in nursing have made a mistake and should instead be studying or working in medicine. In Sales-Mauricio and colleagues’ study [48], 10.5% of respondents stated that their desire to become nurses was met with comments from their social circles such as: “But you’re clever, why don’t you study medicine?”. In Mao and colleagues’ paper [31], the comments made were “You’re a guy. Why nursing? Why not go into policing or something else?”, while Juliff and colleagues [41] recorded comments such as “What’s a guy doing working as a nurse?” and “Didn’t you want to be a doctor?”. Although some study nursing as an indirect route into medicine or paramedicine, others explain that they do not aspire to become doctors but are instead keen to care for and look after people [38].

This stereotype is so deeply rooted in some cultural contexts that patients believe that all men in white uniforms are doctors and all women are nurses [37]. As a result, male nurses are frequently mistaken for doctors, which was a source of embarrassment for participants [37]. On some occasions, they opted not to correct the error as they considered themselves more interesting in this role [36].

### 3.2. Theme 2. Stereotypes of the Nursing Profession

C.9 An Unknown Profession

Ignorance of nursing and nursing professionals within society is a key factor in producing erroneous, inappropriate ideas of the profession [48]. In Woods–Giscombe and colleagues’ study [32], female nurses explained that patients’ expectations of them did not reflect their professional role and that patients neither knew nor understood nurses’ role and tasks. This ignorance was shared by a student nurse, who commented that he had not fully grasped what nurses did until he began to study nursing [32], and by several participants in a reviewed study [30], who were unable to describe what nursing consists of or what nurses do without comparing them to doctors.

In some studies, nurses were described as handling body fluids and other materials, administering injections, and providing patients with basic care [23,32,39,40]. This image has given rise to the belief that nurses do little more than ensure patients’ hygiene and may affect the level of family support for students’ decision to study nursing, with some relatives opposing their choice on the basis that nurses only “wipe bottoms” [48]. Nursing is sometimes perceived as a repetitive, “boring job” [23] or as a “dirty job” [39], of little importance to patients [23]. Nurses’ work was viewed as stressful as they have to deal with distressed patients and relatives, as well as difficult, demanding, laborious, and requiring intense physical effort [31].

With regard to ignorance of the profession, the image of nursing portrayed in the media showed a profession with limited opportunities for growth [32].

C.10 A Valued But Not Prestigious Profession

Sanz Vega and colleagues [46] observed that 97% of participants trusted nurses to enter their homes; 75.6% trusted them to administer a new form of care; and 56.1% trusted nurses to prescribe medication. A participant in Marcinowicz and colleagues’ study [40] expressed the opinion that nurses were highly trusted, much more highly than doctors. The participants in Alexander and colleague’s paper [30] had a positive perception of the care provided by nurses.

Despite these positive assessments, other studies point to the profession’s low prestige and social status [23,40,48]. This affects the degree of acceptance from nurses’ families when they reveal their chosen profession, which some people consider not to be good enough [33].

In some cultural contexts, as in 71% of Elmorshedy and colleagues’ sample, people would feel ashamed to have a nurse in the family [44]. This feeling of shame increased when the nurse was male [23].

The degree of social prestige associated with the profession appears to derive from a comparison with other professions, such as medicine [39]. This factor was expressed by young people seeking to study medicine, who believed that they would obtain greater power and status as doctors than as nurses [30,48]. In Kämmer and colleagues’ study [43], a participant commented that nurses belong to a different social class and are poorer than doctors. However, the participants in another research study rated nursing as the second most valued profession after medicine [46]. In Liaw and colleagues’ paper [39], students attributed the prestige surrounding the medical profession to social media, pointing to the fact that doctors are often portrayed as heroes while nurses are cast in a more passive role. 31.2% of participants in another study disagreed with this portrayal [46].

Meanwhile, in Woods–Giscombe and colleagues’ article [32], students believed that nursing was just as prestigious as medicine, and in Čukljek and collegues’ study [47], they stated that “the service given by nurses is as important as that given by physicians”.

Other studies noted the lack of respect for nurses shown by patients, doctors, and society in general [23,30]. This corroborates the opinions of the third-year students surveyed by Čukljek and colleagues [47], who expressed lower levels of agreement with the item “Nursing is a respected profession” than first-year students, with a statistically significant difference.

C.11 A Subordinate Profession

The autonomy of the nursing profession is called into question in studies such as Alexander and Diefenbach [30], where the participants used terms such as “subordinate” to describe nursing. In another study, the participating students believed that doctors were respected for their ability to make autonomous diagnoses, whereas nurses were subordinate and had to abide by the doctors’ decisions [39]; this was corroborated in several studies [32,39,40,44]. Nursing is perceived as a profession in which doctors’ orders and instructions are obeyed [23,30,35], resulting in acquiescence to doctors and undervaluing of nurses [48]. This vision was expressed by some nursing professionals, who stated that doctors give instructions to nurses and that only doctors must speak in front of patients, with nurses remaining in the background [43]. One male nurse in Kluczyńska and colleagues’ study [38] explained that he struggled to defer to doctors as he was a man, viewing subordination as easier for women.

Other studies presented different conclusions, such as Čukljek and colleagues’ article [47], in which third-year students agreed more strongly with the statement “Nurses are capable of independent practice” than first-year students, with a statistically significant difference. Moreover, 69.1% of the sample in Sanz Vega and colleagues’ paper [46] stated that nurses have their own duties that do not depend on doctors.

C.12 A Profession with Low Academic Requirements

One of the stereotypes of the nursing profession is that it requires a low academic level, which is compared in some studies to the stricter academic requirements in other professions [30]. The academic qualifications required to access nursing are lower than those needed for medicine, making it a good option for students who fail to achieve the necessary grades and do not want to resit their exams [37].

With regard to the type of training received by nurses, 80.8% of the general public participating in Sanz Vega and colleagues’ study [46] identified it as university education and 72.8% were aware that there are nursing specialities. By contrast, one-third of the participants in another study disagreed that nurses need a university education [44].

With regard to training quality, most professionals [49] and members of the general public [46] declared that the nursing profession was highly-skilled. In Čukljek and colleagues’ study [47], the participating students strongly agreed with all the items related to nursing education, including “Nurses with completed undergraduate nursing studies and graduate studies significantly contribute to patient care” and “Nurses integrate health teaching into their practice”. The level of agreement increased among final-year students compared to first-year students. This was the only study to present results on nursing research, noting that participants agreed or strongly agreed with the following statements: “Research is vital to nursing as a profession”; “The major goal of nursing research is to improve patient care”; and “Nurses incorporate research findings into their clinical practice” [47].

C.13 A Profession with Questionable Working Conditions

The participants in two studies [38,49] expressed the belief that nursing provided permanent, stable employment. 77.8% of those surveyed by Chen and colleagues [23], and other studies [39,48] explained that the job opportunities, convenience, and stability offered by the profession had influenced their decision to become nurses. These factors are reflected in: “Nursing provided a safe haven and the opportunity for families entering the country to live the American dream” [30] and in the view that nursing is a good opportunity for women to obtain a secure, stable job [40]. However, in Čukljek and colleagues’ research [47], third-year students strongly agreed with: “Nurses should have a right to strike”, and disagreed with: “Nurses speak out against inadequate working conditions”.

The participants in another study [31] thought that the salary was decent and higher than average. However, it was viewed as insufficient or inadequate [47] and as low in comparison to other healthcare professionals [38,39,49], which may have a negative impact on the prestige assigned to the profession [40].

Several studies pointed to other benefits besides salary: economic stability; having a place to live; health insurance [29,31,38]; and opportunities for promotion within the profession [31,37].

## 4. Discussion

This review revealed two different types of stereotypes: stereotypes relating to the gender identity of nursing professionals, and stereotypes relating to the characteristics of the nursing profession itself.

The stereotypes relating to nursing professionals were shaped by the gender roles imposed on both men and women in contemporary society. Women are assigned a role based on reproduction and care for the family, which are viewed as innate to women’s nature. These characteristics are also attributed to female nurses, who are considered more suitable for the profession than male nurses [50].

Terry and colleagues [19] explain that the feminisation of the nursing profession in educational and professional contexts gives rise to stereotypes that constrain women’s professional activity and limit men’s participation in the profession. Female nurses are valued less as they perform intrinsically female tasks and are not perceived as specialised professionals. The failure to value female nurses’ professional activity has an impact on their everyday work: they receive more verbal insults [51]; their work is sexualised; and they suffer more degrading treatment in the workplace [15]. Some studies add that this discrimination can be observed in the exercise of the profession and in the threat to patriarchal societies posed by women wishing to study and delaying marriage and childbirth [52,53,54,55]. The evidence analysed provides no specific information about the stereotypes assigned to women who decide to enter nursing, although the results obtained for men do reveal this information.

Men experience social judgement over the role they are expected to perform both personally and professionally. They are assigned a productive role, distancing them from elements related to the female role (caring, empathy, physical or emotional proximity, etc.) [9]. Our analysis shows that men who decide to enter the nursing profession face two main types of prejudice: they are viewed as not working in a profession appropriate to men and suspicions are aroused as to their sexual orientation and masculinity [24,25,29,37,41,48]. According to other studies [56,57], men also experience these prejudices in other professions considered “feminine”. Our results reveal that men respond to this discrimination by: deciding not to enter these disciplines, which increases the gender gap and encourages feminisation in certain fields; participating in these disciplines, but aspiring to management or leadership positions; or accepting that they will be subject to discrimination because of their profession. Male representation in positions of responsibility is not unique to nursing and can also be observed in other professions [58] and healthcare disciplines [59,60], where management and leadership positions are held by individuals who are not representative of the overall profession.

Male nurses are not only judged for working in a so-called ‘female’ profession, they are also questioned within the nursing discipline for working in areas such as paediatrics, maternity, and gynaecology [17], in which they are believed to diverge from their natural male role and pose a potential sexual threat to the patients in their care. This sexual threat, viewed as innate to men in the articles analysed [27,33,42], reflects the current social debate on the nature of the male sexual impulse and men’s predisposition to sexually abuse women [61].

The literature analysed also highlighted the prejudices held by members of the nursing profession itself, which include: lower value than other healthcare disciplines; low-skilled profession or subordinate to other healthcare professions; working conditions, such as low pay and professional esteem; and ignorance of the tasks actually performed by nurses. López-Verdugo and colleagues [17] found that this discriminatory vision of the nursing profession derived primarily from the portrayal of the profession in the media, social media, and social information [15]. The value of nursing in society has risen as a result of the response to the COVID-19 pandemic [62,63], but this has not been reflected in nurses’ pay nor in the real value or recognition of their professional activity [64].

The review reveals the current view of stereotypes associated with the nursing profession and its professionals. This allows planning interventions that could change the image attributed, depending on social contexts. It seems that the actions which will generate this change should be aimed at promoting real knowledge of the skills of the profession and the leadership of its professionals. For this purpose, a real image of nursing professionals should be projected in all media, including the functions and areas of work for which they have the competencies and training, and the responsibilities they have for multidisciplinary teams, without gender linkage. The image should show a profession in which men and women can work together and in which the skills, and not the gender of the professional, are those that mark the functions that each one can perform. The sessions and open days, held by the universities for the recruitment of future students, are another opportunity to introduce leading and prestigious nursing professionals, in addition to showing real nursing. Moreover, nursing universities have an important mission because they must promote an excellent acquisition of professional skills among their students, without defined gender. This aspect should be transversal and should be oriented both theoretical and practical teaching. Finally, a real participation of nursing professionals in service management and health policy planning should be encouraged. These are areas where male nurses should not have priority over female nurses. Action can also be taken, from official organizations in the media and education centers, to ensure that not only feminine terms are used to refer to nursing professionals.

This review has a number of limitations. Firstly, backward snowballing was not used to identify other relevant articles. Secondly, the different methodologies used in the selected studies made it difficult to compare their results. Finally, the cultural and geographical differences present in the selected studies may have produced a number of discrepancies between their results, as the topics were cultural constructs.

## 5. Conclusions

Nursing is considered a women’s profession, limiting male participation. It is perceived as a low-skilled, low-prestige, poorly paid profession requiring little academic training, which is easy to enter. Nursing is also viewed as a profession lacking autonomy and subordinate to the medical profession.

The work carried out by nurses is assigned a low value by society as it is regarded as being suitable for women, who are not considered competent for management roles. In some cultural contexts, the decision to become a nurse can lead to delays in starting a family.

Male nurses encounter different stereotypes, which question their masculinity, their control over their sexual impulses, and their professional competence. More patriarchal religious and cultural contexts are associated with harsher stereotypes of male nurses.

The most effective way of reducing stereotypes and bias in the nursing profession is to portray it as a scientific discipline in which both men and women can establish their careers, highlighting the development, level of specialisation, and areas of work involved in the discipline. Appropriate channels for this awareness-raising work include interventions from universities at recruitment days, the public image portrayed in the media, and participation in healthcare policies.

## Figures and Tables

**Table 1:** Search strategies.

Terms	Strategies
1	((stereotyping (MESH)) OR (stereotyping (MH)) OR (stereotyping (TI)) OR (stereotyping (AB)) OR (stereotyping (T/A)))
2	((nursing (MESH)) OR (nursing (T/A)) OR (nursing (TI)) OR (nursing (AB)) OR (nurse (MH)) OR (nurse (T/A)) OR (nurse (TI)) OR (nurse (AB)) OR (nurses (MH)) OR (nurses (T/A)) OR (nurses (TI)) OR (nurses (AB)))
3	1 AND 2

**Table 2:** Summary of the studies included.

Mixed Method Studies					
First Author, Year and Country	Aim	Method and Sample	Data Collection and Analysis	Main Results	Methodological Quality Assessment
Chen, Y., 2020 [23] China	To investigate the changing tendencies and influences on the professional identity of male nursing students in 3-year colleges and junior practising male nurses in China	Mixed methods: non-experimental quantitative, and qualitative study *n* = 237 male nursing students	Professional Identity Questionnaire of Nursing Students and in-depth semi structured interviews Statistical analysis by SPSS and thematic analysis by Braun and Clarke approach	Stereotyped images of male nurses can have a negative impact on their marriage prospects. Nurses provide patients with basic care, which is viewed as laborious, repetitive, and tedious. They are believed to follow doctors’ orders and to do what they are told. Their work is not considered important, they receive little respect from patients, and their social status is low.	S1. Yes S2. Yes 5.1 Yes 5.2 Yes 5.3 Yes 5.4 Yes 5.5 Can’t tell
Kim, I.-J., 2017 [24] South Korea	To investigate and describe South Korean societal perceptions of male nurses	Mixed methods study with Q methodologic step 1, *n =* 7 participants; step 3, *n =* 45 participants, general population	Q method PQMethod program	Progressive perspective (22 participants): they do not perceive nurses as either male or female and hold no gender stereotype. Sympathetic perspective (4): they hold a very positive view of male nurses and are opposed to all gender discrimination. Conservative perspective (6): they hold a stereotypical view of nursing and male nurses; their best quality is that they are strong and sturdy. Negative perspective (4): they hold a negative opinion of both the profession and of male nurses.	S1. Yes S2. Yes 5.1 Yes 5.2 Yes 5.3 Yes 5.4 Yes 5.5 Can’t tell
**Qualitative Studies**					
**First Author, Year** **and Country**	**Aim**	**Method and** **Sample**	**Data Collection** **and Analysis**	**Main Results**	
Kane, D., 2021 [25] Canada	To explore the recruitment and retention of male nursing students currently enrolled in an undergraduate baccalaureate nursing program to capture their lived experiences as men entering the nursing profession	Qualitative descriptive study *n =* 17 male undergraduate nursing students	Interviews, face to face Content analysis	Men are viewed as less skilled at certain tasks in maternity and obstetrics units. Men who work in nursing are perceived as homosexual. The uniforms available are designed for women, leading to conflict among men as they do not have appropriate clothing.	S1. Yes S2. Yes 1.1 Yes 1.2 Yes 1.3 Yes 1.4 Yes 1.5 Yes
Rabie, T., 2021 [26] South Africa	To investigate stereotypes of occupational gender roles about male nurses, as viewed from both emic and etic perspectives.	Qualitative descriptive study *n =* 30; male nurses (*n =* 10) (emic group), female nurses (*n* = 10) and discharged patients (*n* = 10) (etic groups) from four public hospitals.	Semi-structured interviews Thematic analysis using an inductive approach.	Female patients prefer to receive care from women. Male nurses appear not to maintain confidentiality and are often careless and thoughtless when providing basic care, but they are viewed as friendly, helpful, and better at management than women. Male nurses are recognised for their physical strength in particular, and female nurses are given the most important tasks.They feel that they are not provided with clothing appropriate for men.	S1. Yes S2. Yes 1.1 Yes 1.2 Yes 1.3 Yes 1.4 Yes 1.5 Yes
Baker, M.-J., 2021 [27] Australia	To report on the everyday concern of the ‘potential for misinterpretation’, which was the basic social problem revealed in a grounded theory study exploring male nurse practice in inpatient rehabilitation in Australia.	Qualitative Grounded Theory *n =* 38 (23 male nurses and 15 patients (6 women and 9 men)	Observation of practice and semi-structured interviews Methods mapped by Charmaz	Nursing is a women’s occupation. Nursing is not for men. Male nurses are perceived as a sexual threat by men and women. Male nurses are perceived as homosexual.	S1. Yes S2. Yes 1.1 Yes 1.2 Yes 1.3 Yes 1.4 Yes 1.5 Yes
Nogueira, I.-C., 2021 [28] Brazil	To understand the challenges of introducing gender debate in nursing training from undergraduate students’ perspective	Qualitative, exploratory-explanatory study *n =* 12 nursing students	Interviews, face to face Analysis discourse of the collective subject	Nursing is defined as a profession traditionally carried out by women. Men have better leadership and management skills.	S1. Yes S2. Yes 1.1 Yes 1.2 Yes 1.3 Yes 1.4 Yes 1.5 Yes
Saleh, M., 2020 [29] Jordan	To explore the Jordanian male nurses’ experiences of their career within the Arab community	Qualitative hermeneutic and phenomenological study *n =* 22 male nurses	Focus groups Thematic analysis by Van Manen’s perspective.	Patients prefer to receive care from female nurses. Arabs do not hold a positive view of nursing as a professional career for men. Male nurses are not well received in different areas of the hospitals, but are preferred for performing vaious techniques because they are perceived as more competent than female nurses for specific tasks. Administrative positions are provided for men. Women are obliged to care for their families as well as working, which could affect their performance. They felt uncomfortable when people called them “sisters”.	S1. Yes S2. Yes 1.1 Yes 1.2 Yes 1.3 Yes 1.4 Yes 1.5 Yes
Alexander, R.-K., 2020 [30] South Africa	To explore the perceptions of nursing held by African American undergraduate non-nursing science majors within the context of their career ideals.	Qualitative descriptive study *n =* 20 African American non-nursing science majors	Semi-structured interviews Thematic analysis by Miles, Huberman and Saldaña’s coding processes	Positive perceptions of nursing were tempered by concerns about limited respect for the profession and its perceived lack of power. They spoke favourably of nursing care. Most indicated that while they themselves have respect for nurses, that sentiment is not always shared by the public, patients, and doctors. Most believed that careers in medicine would afford them personal and professional power and status. Limited autonomy of nurses and opportunities to personally effect substantive change are confined by the limits of physicians’ supervision, so they do not view nurses as powerful role models.	S1. Yes S2. Yes 1.1 Yes 1.2 Yes 1.3 Yes 1.4 Yes 1.5 Yes
Mao, A., 2020 [31] China	To explore factors influencing the recruitment and retention of male nurses.	Qualitative study *n =* 24 male nurses	Semi-structured interviews Thematic analysis by Smith approach and with +Nvivo11 Plus software	Nursing is not a men’s job and they were ashamed to be nurses. Male nurses are expected to be more specialised. Opinion on usual working conditions (low salary, hard work) versus acceptable working conditions (high/decent salary, stable employment).	S1. Yes S2. Yes 1.1 Yes 1.2 Yes 1.3 Yes 1.4 Yes 1.5 Yes
Woods-Giscombe, C., 2020 [32] United States	The purpose of the current study was to analyse data from previously collected interviews with students who participated in the CaBB program to identify their perspectives on the influence of family, friends and others on nursing as a career choice and, optimal recruitment strategies to enhance diversity in schools of nursing.	Qualitative Theory of Social representation *n =* 22 students who were participants in the CaBB program.	Focus group Thematic analysis by Guest, MacQueen and Namey’s approach	Strong influence of family and media in erroneous perceptions of the profession. There is a lack of knowledge within society and among patients regarding nurses’ role and tasks in caring for patients	S1. Yes S2. Yes 1.1 Yes 1.2 Yes 1.3 Yes 1.4 Yes 1.5 Yes
Jamieson, I., 2019 [33] New Zealand	To describe male nursing students’ understanding of the gender stereotypes associated with nursing.	Qualitative descriptive study *n =* 8 men enrolled in a graduated entry nursing program.	Semi-structured interviews Thematic analysis by Braun and Clarke approach	Nursing is viewed as a women’s job or role. Patients prefer female nurses if they are allowed to choose and feel uncomfortable with male nurses. Society assumes that male nurses are gay or similar. Most of the male nurses were comfortable with the majority of their colleagues being women. Male nurses have a lower status and financial level, and do not meet society’s expectations.	S1. Yes S2. Yes 1.1 Yes 1.2 Yes 1.3 Yes 1.4 Yes 1.5 Yes
Ndou, N.P., 2018 [34] South Africa	To explore and describe 4-year diploma male students’ experiences in a profession traditionally perceived as a female domain	Qualitative descriptive and exploratory study *n =* 30 African male nursing students	Focus group Thematic analysis by Tesch’s step, described by Creswell	The female student nurses were given more opportunities than male student nurses The use of feminine terms was distasteful for them. Male student nurses were asked to perform non-nursing duties. They feel lost and not belonging. Recruitment bias leading to isolation.	S1. Yes S2. Yes 1.1 Yes 1.2 Yes 1.3 Yes 1.4 Yes 1.5 Yes
Cheng, M.L., 2018 [35] Taiwan	To explore the lived experience of novice male nurses when they first enter the workplace	Qualitative descriptive study *n =* 14 male nurses	Face-to-face interviews with more than one follow-up telephone interview per participant Content analysis by Elo and Kyngas	Students believed that nurses can only obey doctors’ orders and that their work is not considered important for patients. In their cultural context, being a male nurse can affect romantic relationships. Men received more favourable treatment from classmates and teachers, both men and women.	S1. Yes S2. Yes 1.1 Yes 1.2 Yes 1.3 Yes 1.4 Yes 1.5 Yes
Liu, H.Y., 2017 [36] Taiwan	To explore the gendered experiences of male nursing students during their first initial nursing clinical practice.	Qualitative Grounded Theory *n =* 22; 10 sophomore male nursing students and 12 sophomore female nursing students. Personal interview 4 males	Focus group and individual face to face interviews	To counter the gender stereotypes associated with their masculinity, male nurses reinforced their masculine identity. As men, they were sometimes assigned the role of doctor and, in some cases, they opted not to correct this. They pointed to the use of feminine terms that perpetuate social perceptions of nursing as a women’s profession and make male nurses feel uncomfortable.	S1. Yes S2. Yes 1.1 Yes 1.2 Yes 1.3 Yes 1.4 Yes 1.5 Yes
Yang, C.I., 2017 [37] China	To investigate how male nursing students in Taiwan perceive the barriers to their experience as nursing students and how they manage these barriers in their study environment and social life.	Qualitative study *n =* 24 male nurses	Semi structured interview suggested by Meadus and Twomey Thematic analysis by Brun and Clarke	The nursing profession is an obstacle to women’s lives when it comes to starting a family. Male nurses are homosexual. Nursing is not a man’s job. Male nurses are viewed as a sexual threat. Male student nurses are less skilled than women, they tend to opt for more technical areas and are ‘vetoed’ from other areas. Male nurses have greater opportunities for career development than women. The advantage of being a male nurse is that they are stronger than women. Nursing offers good job prospects.	S1. Yes S2. Yes 1.1 Yes 1.2 Yes 1.3 Yes 1.4 Yes 1.5 Yes
Kluczyńska, U., 2017 [38] Poland	To establish the main motives for choosing nursing by men in Poland and the results for leaving the profession.	Qualitative research with a grounded theory approach. *n =* 17 licensed male nurses	Individual semi-structured interviews The grounded theory method by Charmaz	Nursing is a women’s profession. Men have difficulty achieving self-fulfilment in the profession as their masculinity is questioned. Nursing is viewed as: a secondary alternative to medicine or paramedicine; a subordinate profession to medicine, which is therefore easier for women; low-paid but stable income; improving healthcare knowledge; enabling self-fulfilment; and valued by society.	S1. Yes S2. Yes 1.1 Yes 1.2 Yes 1.3 Yes 1.4 Yes 1.5 Yes
Liaw, S.Y., 2016 [39] Singapore	To identify factors influencing healthcare career choices among Singaporean students and to determine the deterrents in joining the nursing profession	Qualitative, exploratory and descriptive study *n =* 60; 30 nursing students and 30 non-nursing students	Focus group Thematic analysis by Braun and Clarke	Students believe that medicine is a prestigious profession, with doctors portrayed on social media as heroes and nurses attributed a more passive role. They thought that doctors were respected for their ability to issue autonomous diagnoses, whereas nurses were subordinate and had to abide by the doctors’ decisions. They explained that the job opportunities, convenience, and stability offered by the profession had influenced their decision to become nurses.	S1. Yes S2. Yes 1.1 Yes 1.2 Yes 1.3 Yes 1.4 Yes 1.5 Yes
Marcinowiczm L., 2016 [40] Poland	To conduct an in-depth analysis to understand how Polish nurses perceive their profession from the perspective of the jobs they perform and their experiences connected with the work.	Qualitative, descriptive and exploratory study *n =* 60 nurses	Focus group Thematic analysis by Graneheim and Lundman	Nursing is considered an appropriate, positive profession for women. They believed that society sees nurses as administering injections and performing tasks relating to hygiene and ‘medical’ documentation. They detected attitudes that favoured male nurses, who were given more opportunities because they were men. They thought that the salary was low and that was why patients did not respect them. Nurses were highly trusted, much more highly than doctors. They pointed to the low prestige and social recognition associated with the profession.	S1. Yes S2. Yes 1.1 Yes 1.2 Yes 1.3 Yes 1.4 Yes 1.5 Yes
Juliff, D., 2016 [41] Australia	To provide an account of the first phase of a qualitative longitudinal study that explored the initial challenges men in nursing face to become registered	Qualitative longitudinal study *n =* 9 newly graduated male registered nurses	Individual face-to-face interviews by phenomenological approach The analysis process was made via iterative stages	Nursing is perceived as a women’s profession. Nursing is not a job for men, provoking shame among male nurses. Male nurses are viewed as: homosexual; a sexual threat, so contact with women is avoided when personal care is required; unable to express feelings. Male nurses prefer more technical specialities and areas. They felt isolated as they were in a minority. They felt that they were perceived differently to female nurses. Hostility towards male nurses.	S1. Yes S2. Yes 1.1 Yes 1.2 Yes 1.3 Yes 1.4 Yes 1.5 Yes
Cottingham, M., 2016 [42] United States	To explore how men, regardless of their own sexuality, distance themselves from the stereotype of the gay, effeminate nurse, while also grappling with the implications that hypersexual assumptions have for providing intimate care.	Qualitative and interpretive study *n =* 40 female nurses completed daily audio and 35 participated in interviews	Semi-structured interviews Thematic analysis by Hesse-Biber and Laevy	Nursing is a women’s occupation. Male nurses are viewed as homosexual; a sexual threat; and keen to avoid expressing their feelings. Men are dubious care providers. Their ability to deliver care is limited. Another perspective views men as natural carers who are sensitive and nurturing, are not all homosexual, and meet the requirements of the profession.	S1. Yes S2. Yes 1.1 Yes 1.2 Yes 1.3 Yes 1.4 Yes 1.5 Yes
**Quantitative Studies**					
**First Author, Year** **and Country**	**Aim**	**Method and** **Sample**	**Data Collection** **and Analysis**	**Main Results**	
Kämmer, J.E., 2021 [43] Germany	The aim of this study is to inform IPE developers of the prevalence and content of interprofessional stereotypes in the workplace in Germany and similarly structured healthcare systems.	Quantitative study *n =* 129 (97 nurses of both sexes and 32 occupational therapists)	Online survey Statistical analysis by R Core Team e IBM SPSS v24.0.	Nurses are essential, competent, and dedicated to their work. Nursing is viewed as a subordinate profession requiring fewer skills than medicine. Differentiation by social class based on salary; compared to doctors, nurses are poorer. Society values personal qualities over professional qualities in nurses.	S1. Yes S2. Yes 4.1 Yes 4.2 Yes 4.3 Yes 4.4 Can’t tell 4.5 Yes
Elmorshedy, H., 2020 [44] Saudi Arabia	To explore the level of community awareness and public image of the nursing profession in Saudi Arabia.	Quantitative cross-sectional study *n =* 502 university students enrolled in non-health college (106 males/396 females)	Ad-hoc, self-report survey Quantitative statistical analysis by SPSS	Half of sample would like to marry a female nurse. 65% maintained that being a nurse had a negative impact on women’s social lives. For 25%, it was the reason why women delayed marriage. 71% would feel ashamed to have a nurse in the family. 61% perceived nurses as subordinate to doctors. 68.9% disagreed with female nurses holding senior management roles. 33.3% disagreed with nursing being a university degree.	S1. Yes S2. Yes 4.1 Yes 4.2 Yes 4.3 Yes 4.4 Can’t tell 4.5 Yes
Carlsson, M., 2020 [45] Sweden	To describe and compare the self-reported competence in female and male nursing students.	Quantitative cross-sectional study with two points in time 2012 and 2017 *n =* 1810 nursing students	Nursing Professional Competence Scale Quantitative statistical analysis	Items where female students assessed themselves higher than male students: Handle basic physical nursing care; Make sure the patient understands the information; Use of relevant patient record; Handle sensitive information; Pay attention to work-related risks and prevention; Engage in own personal competence development; Lead the team and coordinate nursing care based on the patients’ needs. Items where male students assessed themselves higher than female students: Manage drugs, applying knowledge of pharmacology; Handle medical products; Apply emergency medical principles; Implement new knowledge; Cooperate with other factors; Teach, supervise and assess students; Supervise and train co-workers/staff.	S1. Yes S2. Yes 4.1 Yes 4.2 Yes 4.3 Yes 4.4 Yes 4.5 Yes
Sanz Vega, C., 2020 [46] Spain	To determine the social image of nursing in the Asturian population	Quantitative descriptive and multicentre study *n =* 335 Asturian participants	Ad-hoc, self-report survey Quantitative statistical analysis	Nursing was viewed as a women’s job and people preferred to receive care from a female nurse. It was viewed as the second best profession after medicine, with 73.4% of the sample considering it a good occupation. Nurses perform tasks that do not depend on doctors: strongly agree/agree 69.1%; 10.7% disagree, as all their activities are based on doctors’ orders. 79.1% viewed nursing education as adequate; 80.8% thought that nursing was a university degree. 72.8% believed it was necessary to have nursing specialities. 63.9% did not view marrying a doctor as a benefit of being a nurse.	S1. Yes S2. Yes 4.1 Yes 4.2 Yes 4.3 Yes 4.4 Can’t tell 4.5 Yes
Čukljek, S., 2017 [47] Croatia	To determine the attitudes of nursing students towards nursing, and changes in their attitudes during the study	Quantitative study with pre-post survey *n =* 115 nursing first year students	Survey. Demographic information and Nursing Image Questionnaire translated into Croatian. Developed by Toth et al. Quantitative statistical analysis by SPSS	Agree: One advantage of being a nurse is not to marry a physician/Men make good nurses/The service provided by nurses is as important as that provided by physicians/Nurses incorporate health education into their practice/Research is vital to nursing as a profession/Nurses incorporate research findings into their clinical practice/ Nurses are capable of independent practice/Nurses are not adequately paid for the work they do. Neither agree nor disagree: Many nurses who pursue advanced degrees in nursing would not prefer to be physicians/The main goal of nursing research is to improve patient care/Nurses do not follow physicians’ orders unquestioningly/Nurses speak out against inadequate working conditions/Nurses are not compensated sufficiently for their work given that they are helping people.	S1. Yes S2. Yes 4.1 Yes 4.2 Yes 4.3 Yes 4.4 Yes 4.5 Yes
Sales-Mauricio, L.F., 2016 [48] Brazil	To verify the presence of psychic suffering in male students of the nursing graduation related to gender and, to analyse determining factors and attitudes to cope with psychic suffering	Quantitative descriptive and exploratory study *n =* 16 male nursing students	Ad-hoc, self-report survey Quantitative statistical analysis by SPSS	Nursing is viewed as a profession for women. Participants believed that women are responsible for care due to their maternal role (34.2%) and that nursing education is appropriate (62.5%). Participants were not supported or received offensive messages when they chose to be male nurses. There are areas of care delivery were men should not be present. Patients sometimes refused to allow male nurses. There is an assumption that all men studying nursing are homosexual (15.4%). Men are valued only in situations requiring physical strength. They felt disconcerted and struggled to coexist with so many women (21.4%); they felt different and in a minority (14.4%). Lack of knowledge of nursing and undervaluing by society (9.3%). Subordination to doctors, lack of knowledge of the profession; low levels of recognition within society and team; low pay.	S1. Yes S2. Yes 4.1 Yes 4.2 Yes 4.3 Yes 4.4 Can’t tell 4.5 Yes
Stanley, D., 2016 [49] Australia	To establish a profile of men in nursing in Western Australia and gather information about how men in nursing perceive themselves and are perceived by their female colleagues.	Quantitative, non-experimental, comparative and descriptive study *n =* 1055 nurses and midwives	Survey based in questionnaire Hodes Research and Bartfay Statistical analysis by SPSS 21	The participating men and women viewed nursing as a women’s profession, which was appropriate for them but also for men. Men are recognised more for their physical strength than for their professionalism. They are believed to be better suited to management and leadership, with a preference for more technical areas. The association of the profession with homosexuality dissuaded men from choosing to go into nursing. The profession was highly skilled and offered permanent, stable work, but the salary is low when compared to other healthcare professionals, and this was linked to the level of prestige associated with the profession.	S1. Yes S2. Yes 4.1 Yes 4.2 Yes 4.3 Yes 4.4 Yes 4.5 Yes

**Table 3:** Themes, subthemes and categories of the qualitative analysis.

Themes	Subthemes	Categories
1. Stereotypes relating to professionals’ gender	1.1. Stereotypes of nurses	C.1 Female nurses: the ideal gender for nursing
		C.2 Female nurses: family struggles
	1.2 Stereotypes of male nurses	C.3 Male nurses: out of place
		C.4 Male nurses: male weakness or homosexuality
		C.5 Male nurses: a sexual threat
		C.6 Male nurses: shame and embarrassment
		C.7 Male nurses: physical strength
		C.8 Male nurses: should be doctors
2. Stereotypes of the nursing profession		C.9 An unknown profession
		C.10 A valued but not prestigious profession
		C.11 A subordinate profession
		C.12 A profession with low academic requirements
		C.13 A profession with questionable working conditions

## References

[1] Ellemers N. (2018). Gender Stereotypes. Annu. Rev. Psychol..

[2] Haines E.L., Deaux K., Lofaro N. (2016). The Times They Are a-Changing … or Are They Not? A Comparison of Gender Stereotypes, 1983–2014. Psychol. Women Q..

[3] Kite M.E., Deaux K., Haines E.L. (2008). Gender stereotypes. Psychology of Women: A Handbook of Issues and Theories.

[4] Ramos M.D.B., Riera J.R.M., González G.M. (2010). Gender attitudes and stereotypes in nursing. Cult. Cuid..

[5] Du H., Xiao Y., Zhao L. (2021). Education and gender role attitudes. J. Popul. Econ..

[6] Ward L.M., Grower P. (2020). Media and the Development of Gender Role Stereotypes. Annu. Rev. Dev. Psychol..

[7] Gimeno M.D.C.M., Rodríguez R.C., Ferrer B.M. (2019). Stereotypes, gender roles and chain of care. Transformations in women’s migratory process. Collect. Rev. Cienc. Soc..

[8] Rice L., Barth J.M. (2017). A Tale of Two Gender Roles: The Effects of Implicit and Explicit Gender Role Traditionalism and Occupational Stereotype on Hiring Decisions. Gend. Issues.

[9] Rodríguez A.S. (2018). From the pink cup to the object. Around gender building. Econ. Creat..

[10] Cuddy A.J., Glick P., Beninger A. (2011). The dynamics of warmth and competence judgments, and their outcomes in organizations. Res. Organ. Behav..

[11] Arif S., Khokhar S. (2017). A historical glance: Challenges for male nurses. J. Pak. Med. Assoc..

[12] World Health Organization (2019). Delivered by Women, Led by Men: A Gender and Equity Analysis of the Global Health and Social Workforce.

[13] Aranda M., Castillo-Mayén M.D.R., Montes-Berges B. (2015). Has Changed the Traditional Social Perception on Nurses? Attribution of Stereotypes and Gender Roles. Acción Psicol..

[14] Hoeve Y.T., Jansen G., Roodbol P. (2013). The nursing profession: Public image, self-concept and professional identity. J. Adv. Nurs..

[15] Girvin J., Jackson D., Hutchinson M. (2016). Contemporary public perceptions of nursing: A systematic review and narrative synthesis of the international research evidence. J. Nurs. Manag..

[16] Glerean N., Hupli M., Talman K., Haavisto E. (2017). Young peoples’ perceptions of the nursing profession: An integrative review. Nurse Educ. Today.

[17] López-Verdugo M., Ponce-Blandón J., López-Narbona F., Romero-Castillo R., Guerra-Martín M. (2021). Social Image of Nursing. An Integrative Review about a Yet Unknown Profession. Nurs. Rep..

[18] De Souza R. (2017). Review: Nurses’ views on the impact of mass media on the public perception of nursing and nurse–Service user interactions. J. Res. Nurs..

[19] Terry D., Peck B., Carden C., Perkins A., Smith A. (2020). Traversing the Funambulist’s Fine Line between Nursing and Male Identity: A Systematic Review of the Factors that Influence Men as They Seek to Navigate the Nursing Profession. Eur. J. Investig. Health Psychol. Educ..

[20] Smith B.W., Rojo J., Everett B., Montayre J., Sierra J., Salamonson Y. (2021). Professional success of men in the nursing workforce: An integrative review. J. Nurs. Manag..

[21] Whittemore R., Knafl K. (2005). The integrative review: Updated methodology. J. Adv. Nurs..

[22] Hong Q., Pluye P., Fábregues S., Bartlett G., Boardman F., Cargo M., Dagenais P., Gagnon M.-P., Griffiths F., Nicolau B. (2018). Mixed Methods Appraisal Tool (MMAT); Version 2018. http://mixedmethodsappraisaltoolpublic.pbworks.com/w/file/fetch/127916259/MMAT_2018_criteria-manual_2018-08-01_ENG.pdf.

[23] Chen Y., Zhang Y., Jin R. (2020). Professional Identity of Male Nursing Students in 3-Year Colleges and Junior Male Nurses in China. Am. J. Men’s Health.

[24] Kim I.-J., Kim S.-H., Sohn S.-K. (2016). Societal perceptions of male nurses in South Korea: A Q-methodological study. Jpn. J. Nurs. Sci..

[25] Kane D., Rajacich D., Andary C. (2020). Exploring the Contextual Factors Surrounding the Recruitment and Retention of Men in a Baccalaureate Nursing Program. Nurs. Forum.

[26] Rabie T., Rossouw L., Machobane B.F. (2020). Exploring occupational gender-role stereotypes of male nurses: A South African study. Int. J. Nurs. Pract..

[27] Baker M.J., Fisher M.J., Pryor J. (2021). Potential for misinterpretation: An everyday problem male nurses encounter in inpatient rehabilitation. Int. J. Nurs. Pract..

[28] Nogueira I.C., Santos D.D.S., Sanfelice C.F.D.O., Silva E.M., Assis A.E.S.Q. (2021). Gender debate as a challenge in nursing training. Rev. Bras. Enferm..

[29] Saleh M.Y., Al-Amer R., Al Ashram S.R., Dawani H., Randall S. (2020). Exploring the lived experience of Jordanian male nurses: A phenomenological study. Nurs. Outlook.

[30] Alexander R.K., Diefenbeck C. (2020). Challenging stereotypes: A glimpse into nursing’s difficulty recruiting African Americans. J. Prof. Nurs..

[31] Mao A., Wang J., Zhang Y., Cheong P.L., Van I.K., Tam H.L. (2020). Factors influencing recruitment and retention of male nurses in Macau and mainland China: A collaborative, qualitative study. BMC Nurs..

[32] Woods-Giscombe C.L., Rowsey P.J., Kneipp S., Lackey C., Bravo L. (2020). Student perspectives on recruiting underrepresented ethnic minority students to nursing: Enhancing outreach, engaging family, and correcting misconceptions. J. Prof. Nurs..

[33] Jamieson I., Harding T., Withington J., Hudson D. (2019). Men entering nursing: Has anything changed?. Nurs. Prax. N. Z..

[34] Ndou N.P., Moloko-Phiri S.S. (2018). Four-year diploma male students’ experiences in a profession traditionally perceived as a female domain at a selected public college of nursing in Limpopo, South Africa. Curationis.

[35] Cheng M.-L., Tseng Y.-H., Hodges E., Chou F.-H. (2016). Lived Experiences of Novice Male Nurses in Taiwan. J. Transcult. Nurs..

[36] Liu H.-Y., Li Y.L. (2017). Crossing the gender boundaries: The gender experiences of male nursing students in initial nursing clinical practice in Taiwan. Nurse Educ. Today.

[37] Yang C.-I., Yu H.-Y., Chin Y., Lee L.-H. (2017). There is nothing wrong with being a nurse: The experiences of male nursing students in Taiwan. Jpn. J. Nurs. Sci..

[38] Kluczyńska U. (2017). Motives for choosing and resigning from nursing by men and the definition of masculinity: A qualitative study. J. Adv. Nurs..

[39] Liaw S.Y., Wu L.T., Holroyd E., Wang W., Lopez V., Lim S., Chow Y.L. (2016). Why not nursing? Factors influencing healthcare career choice among Singaporean students. Int. Nurs. Rev..

[40] Marcinowicz L., Owlasiuk A., Perkowska E. (2016). Exploring the ways experienced nurses in Poland view their profession: A focus group study. Int. Nurs. Rev..

[41] Juliff D., Russell K., Bulsara C. (2016). Male or Nurse what comes first?: Challenges men face on their journey to nurse registration. Aust. J. Adv. Nurs..

[42] Cottingham M.D., Johnson A., Taylor T. (2016). Heteronormative Labour: Conflicting Accountability Structures among Men in Nursing. Gend. Work. Organ..

[43] Kämmer J.E., Ewers M. (2021). Stereotypes of experienced health professionals in an interprofessional context: Results from a cross-sectional survey in Germany. J. Interprof. Care.

[44] Elmorshedy H., AlAmrani A., Hassan M.H.A., Fayed A., Albrecht S.A. (2020). Contemporary public image of the nursing profession in Saudi Arabia. BMC Nurs..

[45] Carlsson M. (2020). Self-reported competence in female and male nursing students in the light of theories of hegemonic masculinity and femininity. J. Adv. Nurs..

[46] Sanz-Vega C.M., Martínez-Espinosa A., Longo-Alonso C., Charro-Alonso S., Antón-Martínez G., Losada-Riesgo V.C. (2020). Una fotografía de la imagen social de la Enfermería. RqR Enfermería Comunitaria.

[47] Čukljek S., Jureša V., Bile C.G., Režek B. (2017). Changes in Nursing Students’ Attitudes Towards Nursing During Undergraduate Study. Acta Clin. Croat..

[48] Mauricio L.F.S., Marcolan J.F. (2016). The male being in psychic suffering in the nursing course. J. Nurs. UFPE Line.

[49] Stanley D., Beament T., Falconer D., Haigh M., Saunders R., Stanley K., Wall P., Nielson S. (2016). The male of the species: A profile of men in nursing. J. Adv. Nurs..

[50] Hoyle L.P., Kyle R.G., Mahoney C. (2017). Nurses’ views on the impact of mass media on the public perception of nursing and nurse–Service user interactions. J. Res. Nurs..

[51] Gauci P., Peters K., O’Reilly K., Elmir R. (2021). The experience of workplace gender discrimination for women registered nurses: A qualitative study. J. Adv. Nurs..

[52] Alharbi M., McKenna L., Whittall D. (2019). Social barriers experienced by female Saudi nursing students while studying nursing: A phenomenological study. Nurse Educ. Pract..

[53] Cho S., Jang S.J. (2021). Do Gender Role Stereotypes and Patriarchal Culture Affect Nursing Students’ Major Satisfaction?. Int. J. Environ. Res. Public Health.

[54] Almutairi A.F., McCarthy A., Gardner G.E. (2015). Understanding Cultural Competence in a Multicultural Nursing Workforce: Registered nurses’ experience in Saudi Arabia. J. Transcult. Nurs..

[55] Price S.L., Hall L.M. (2013). The history of nurse imagery and the implications for recruitment: A discussion paper. J. Adv. Nurs..

[56] Viana H.A., Torres A.R.R., Estriamana J.L. (2020). Egalitarian men: Stereotypes and discrimination in the labor market. Acta Colomb. Psicol..

[57] Durin S. (2013). Males in Domestic Service in the Monterrey Metropolitan Area: Gender Ideologies in Organizing Work. Trayectorias.

[58] Bompolaki D., Pokala S.V., Koka S. (2021). Gender diversity and senior leadership in academic dentistry: Female representation at the dean position in the United States. J. Dent. Educ..

[59] Garstka M.E., Randolph G.W., Haddad A.B., Nathan C.O., Ibraheem K., Farag M., Deot N., Adib H., Hoof M., French K. (2019). Gender disparities are present in academic rank and leadership positions despite overall equivalence in research productivity indices among senior members of American Head and Neck Society (AHNS) Fellowship Faculty. Head Neck.

[60] Seo G., Huang W., Han S.-H.C. (2017). Conceptual Review of Underrepresentation of Women in Senior Leadership Positions from a Perspective of Gendered Social Status in the Workplace: Implication for HRD Research and Practice. Hum. Resour. Dev. Rev..

[61] Christensen L.S., Darling A.J. (2020). Sexual abuse by educators: A comparison between male and female teachers who sexually abuse students. J. Sex. Aggress..

[62] Rojas-Ocaña M., Araujo-Hernández M., Romero-Castillo R., Román-Mata S., García-Navarro E. (2020). Nursing as a Sustainability Factor of the Health System during the COVID-19 Pandemic: A Qualitative Study. Sustainability.

[63] Williams G., Gunn A., Sweeny A. (2021). Nursing in the time of COVID-19: Exploring nurse preparedness early in a global pandemic. Aust. J. Adv. Nurs..

[64] Greene J., El-Banna M.M., Briggs L.A., Park J. (2017). Gender differences in nurse practitioner salaries. J. Am. Assoc. Nurse Pract..

